# The impact of faecal diversion on the gut microbiome: a systematic review

**DOI:** 10.1017/gmb.2024.1

**Published:** 2024-02-19

**Authors:** Shien Wenn Sam, Bilal Hafeez, Hwa Ian Ong, Sonia Gill, Olivia Smibert, Aonghus Lavelle, Adele Burgess, David Proud, Helen Mohan

**Affiliations:** 1 Faculty of Medical and Health Sciences, University of Melbourne, Parkville, VIC, Australia; 2 Department of Surgery, Austin Health Department of Surgery, Heidelberg, VIC, Australia; 3 Department of Surgery, Peter MacCallum Cancer Centre, Melbourne, VIC, Australia; 4 Department of Anatomy & Neuroscience and APC Microbiome Ireland, University College Cork, Cork, Ireland

**Keywords:** diversion, ileostomy, colostomy, stoma, microbiome, microbiota, dysbiosis

## Abstract

Diversion of the faecal stream is associated with diversion colitis (DC). Preliminary studies indicate that microbiome dysbiosis contributes to its development and potentially treatment. This review aims to characterise these changes in the context of faecal diversion and identify their clinical impact. A systematic search was conducted using MEDLINE, EMBASE and CENTRAL databases using a predefined search strategy identifying studies investigating changes in microbiome following diversion. Findings reported according to PRISMA guidelines. Of 743 results, 6 met inclusion criteria. Five reported significantly decreased microbiome diversity in the diverted colon. At phylum level, decreases in Bacillota with a concomitant increase in Pseudomonadota were observed, consistent with dysbiosis. At genus level, studies reported decreases in beneficial lactic acid bacteria which produce short-chain fatty acid (SCFA), which inversely correlated with disease severity. Significant losses in commensals were also noted. These changes were seen to be partially reversible with restoration of bowel continuity. Changes within the microbiome were reflected by histopathological findings suggestive of intestinal dysfunction. Faecal diversion is associated with dysbiosis in the diverted colon which may have clinical implications. This is reflected in loss of microbiome diversity, increases in potentially pathogenic-associated phyla and reduction in SCFA-producing and commensal bacteria.

## Introduction

The human microbiome is a complex ecosystem of bacteria, archaea, viruses, and eukarya found virtually along every surface of the human body (Shreiner et al., 2015; Berg et al., 2020; Ferrie et al., 2020). Microbiomes are key contributors to health and disease via important host–microbiota interactions (Shreiner et al., 2015). The recent introduction of culture-independent analytical techniques, from metagenomics to metabolomics has made detailed study of the microbiome possible (Shreiner et al., 2015).

The gut microbiome is crucial for intestinal health maintenance, and its role in nutrition-related, metabolic and inflammatory disorders has previously been established (Doré et al., 2013; Shreiner et al., 2015; Ferrie et al., 2020). Diversity and richness of microbiome increases with distal progression along the gastrointestinal tract (GIT), although this varies greatly between and within individuals (Shreiner et al., 2015; Ferrie et al., 2020). The volume of colonic microbiota exceeds that of all other organs by at least two orders of magnitude (Sender et al., 2016) and is chiefly implicated in discussions concerning the “gut microbiome” (Shreiner et al., 2015).

The gut microbiome is sensitive to environmental changes such as diet, smoking, antibiotics, and even gastrointestinal surgery (Shreiner et al., 2015; Rolhion and Chassaing, 2016; Valdes et al., 2018; Ferrie et al., 2020). In this context, maintenance in microbiome diversity may protect against these changes by providing stability, with a reduction in diversity often associated with pathological conditions such as inflammatory bowel disease and infectious colitis in the case of *C. difficile* (Ferrie et al., 2020).

Recent large trials such as the Rotterdam Study (RSIII) approximated the colonic microbiome via faecal studies, showing the dominant phyla as Bacillota (77.8%) and Bacteroidia (12.5%), with lesser extents of Pseudomonadota (4.9%) and Actinomycetota (4.1%). These findings are consistent with other similar studies (Zhernakova et al., 2016; Deschasaux et al., 2018). Recent results from the Dutch Microbiome Project have suggested that individual environmental factors contribute significantly to the interindividual variability of the microbiome (Gacesa et al., 2022).

No singular definition of a healthy microbiota exists, due in part to the heterogeneity of existing studies, but also because of the huge variance within the human microbiome, which has yet to be fully accounted for (Lightner and Pemberton, 2017). One way to define health, as seen with the Dutch Microbiome Project, is to correlate patterns of bacterial presence, recognised as “signatures” of health, with disease and medication use (Gacesa et al., 2022). In other studies, low levels of specific bacteria such as Pseudomonadota combined with abundance of signature SCFA-producing genera from the other three phyla such as *Bacteroidia*, *Ruminococcus*, *Lactobacillus* and *Bifidobacterium* generally indicate a functional colonic environment in homeostasis (Shreiner et al., 2015).

Faecal diversion involves creation of an ostomy (typically ileostomy or colostomy) to divert the faecal stream from the distal end of the GIT (Remzi, 2017). This is most commonly performed following a low anterior resection for rectal cancer, particularly after radiotherapy, or acute colonic resections where inflammation or infection increases the risk of anastomotic leak,. Faecal diversion can be temporary or permanent, and is designed to mitigate the risk of severe sepsis in the event of an anastomotic leak. Diversion without resection may also be performed in severe perianal fistulising disease to promote perianal healing by preventing lesion-to-stool contact, such as in Crohn’s disease (CD) (Whelan et al., 1994; Remzi, 2017).

The stoma results in a functional end that receives nutrients from the faecal stream and a defunctioned end which does not. The diverted or defunctioned end is at high risk of diversion colitis (DC) (~70-90% by various estimates) (Ten Hove et al., 2018; Pieniowski et al., 2020). Treatment may involve stoma reversal, which often improves symptoms; however, these patients are then at increased risk of developing lower anterior resection syndrome (LARS) and *C. difficile* colitis (~18-55%, and ~1-4%, respectively) (Harries et al., 2017; Dou et al., 2020). While the precise pathophysiology is unclear, limited preliminary evidence suggests that colonic microbiome alterations due to diversion may be a contributing factor.

In murine models, oral short-chain fatty acids (SCFA) has been used successfully to treat various forms of murine colitis via restoration of gut microbiota–host interactions (Harig et al., 1989). In humans, faecal microbial transplants (FMTs) have also been effectively employed to treat recurrent *C. difficile* colitis while SCFA enemas have also shown limited success at reducing symptoms of DC patients (Rao and Safdar, 2015; Radjabzadeh et al., 2020). We therefore hypothesise that loss of enteral nutrition in the diverted colon results in dysbiosis, especially of SCFA-producing microorganisms, consequently impacting intestinal structure, function and immunity leading to increased risk of inflammation and disease. Understanding the colonic microbiome changes that occur in the context of diversion may thus be key in characterising and managing these adverse outcomes.

Given the potential relevance of the microbiome in dysbiosis outcomes following diversion, there is a need to understand microbiome changes in the diverted colon. Recent studies are few and heterogenous. Therefore, we seek to systematically review the existing literature, identify key knowledge gaps and highlight areas requiring further attention.

Our aims are to: firstly, characterise the longitudinal changes in the colonic microbiome that occur post-diversion, and secondly, to identify microbiome characteristics associated with dysbiosis related outcomes post-diversion.

## Methodology

### Search strategy

**Figure 1.**
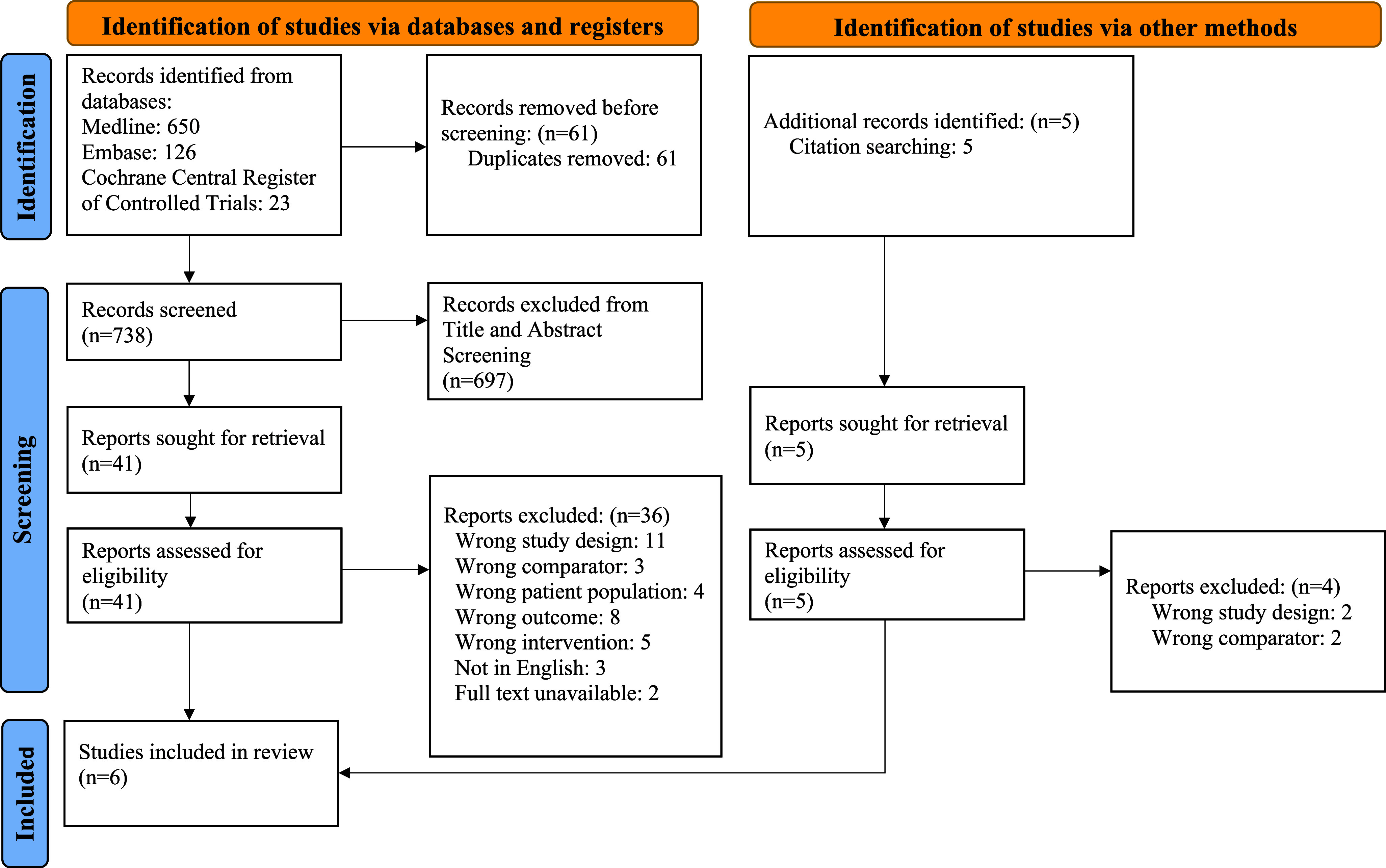
Prisma diagram.

**Figure 2.**
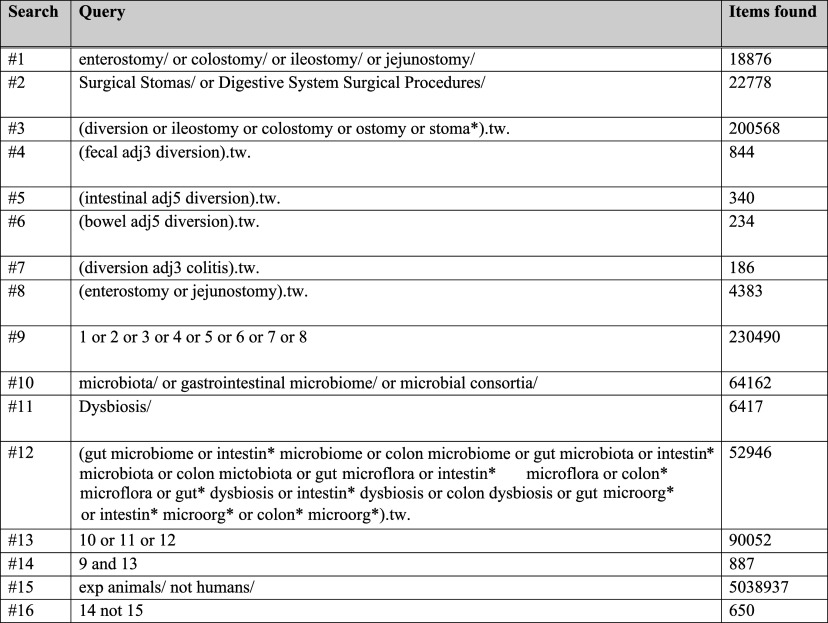
Search strategy for MEDLINE (22 Jul 2022).

A systematic search was designed according to PRISMA guidelines (Figure 1). The search strategy involved searching combinations of keywords and MeSH terms related to 2 key concepts – diversion and microbiome – in the MEDLINE, EMBASE and CENTRAL databases. An example of the MEDLINE search is shown in Figure 2 and adapted as required for EMBASE and CENTRAL, respectively. We also performed secondary backward and forward citation searching on all included papers as well as potentially relevant reviews. Two independent reviewers conducted screening, inclusion and data extraction, with disputes settled by discussion or with a third independent reviewer if consensus was not reached.

### Inclusion and appraisal

We included studies involving adult participants >18y that underwent faecal stream diversion – defined as an ileostomy or colostomy – with measured outcomes that included microbiome analysis on the defunctioned colon post-diversion. Studies that examined microbiome differences pre- and post-diversion, or between functional and defunctioned mucosa *in the* same individuals were included, as well as those that utilised external controls. While this was not ideal, we believe conclusions within the studies were still informative and valid given the broadly identifiable microbiome trends in healthy external controls.

Paediatric populations were excluded due to their different microbiome composition (Joanna Briggs Institute, 2022). Diversion above the jejunum such as biliopancreatic diversion was also excluded as these procedures are not typically associated with colonic dysbiosis outcomes investigated here. Animal-related, non-English, non-full text articles and studies preceding 1998 were also excluded.

Quality and bias assessment was subsequently done on all included papers using the JBI Appraisal tool (Joanna Briggs Institute, 2022). Using the JBI tool, a scoring system similar to Ferrie et al. was used (Ferrie et al., 2020). 1 point was assigned for “Yes” or “NA,” 0 points for “No” and 0.5 for “Unclear” (Table 1).

**Table 1.** Characteristics of studies: Population demographics

References	Study type	JBI composite score	Population			Indication and intervention	
			Sample size^1^	Average age	Gender (Male: Female)	Type of intervention	Indication
Beamish et al. (2017)	Cohort	10/11	34	58 ± 16	17:17	Loop Ileostomy Reversal	Colorectal CA
Tominaga et al. (2021a)	Non-randomised controlled study	9/13^2^	5	65 ± 8	0:5	Loop Colostomy	Colorectal CA Ovarian CA Rectovaginal CA Retroperitoneal Abscess
Tominaga et al. (2021b)	Cohort	8.5/11	8	66 ± 10	1:7	Loop Colostomy (5) End Colostomy (3)	Colorectal CA Ovarian CA Rectovaginal CA Retroperitoneal Abscess
Baek et al. (2014)	Case–control	8/10	26	64 ± 11	15:11	Loop Ileostomy Reversal	Colorectal CA
Young et al. (2013)	Case–control	9.5/10	4	30	4:0	Loop Ileostomy followed by IPAA	Ulcerative Colitis
Watanabe et al. (2021)	Case–control	6.5/10	18 total Cases (CD):6 Controls (CRC):12	Cases (CD): 30 Controls (CRC): 65	Cases (CD): 4:2 Controls (CRC): 6:6	Loop Ileostomy followed by reversal	Crohn’s Disease Colorectal CA

Table 1 showing population demographics for included studies. Only relevant data extracted. In studies investigating multiple interventions or multiple comparison groups, only data directly pertaining to the impact of diversion was extracted. In Watanabe et. al. for example, both case and control groups were reported as both groups received faecal diversion.

Nb: Tominaga et al. (2021a, 2021b) had an overlap of 5 similar patients. Both were, however, different studies altogether – differing in comparisons used, outcomes measured etc. (See Table 2 for full detail) Both were therefore included and treated separately.

CA, cancer; CD, Crohn’s disease; IPAA, ileal pouch anal anastomosis; UC, ulcerative colitis.

1 Control group numbers not added to sample size unless explicitly stated. Only patients who underwent faecal diversion included.

2 JBI tools do not include an NRS checklist, so RCT checklist was used and NA (1 point) assigned to non-applicable criteria.

### Study selection

The summary process and exclusion reasons are shown in full in the PRISMA diagram (Figure 1). The review is reported in keeping with PRISMA guidelines.

## Results

### Included studies

The primary search was conducted on 22/7/22 and identified 738 records after duplicate removal. Following title and abstract screening, Forty-two articles were appraised in full. Five additional articles were further identified during secondary searching and appraised. Six articles were included in the final review.

### Characteristics of included studies

We included 3 case–control, 2 cohort and 1 non-randomised controlled study involving 95 (47m:48f) patients in total, who were generally older in age (>55y) (Table 1). Broadly speaking, most of the studies were small (n<35) and involved diversion procedures in relation to malignancy or IBD. Most of the patients sampled underwent loop ileostomies (n=82), while others had loop (n=10) and end (n=3) colostomies. Apart from this, the studies were heterogenous with regards to sampling and analysis methods, as well as comparators (Table 2). Three studies each utilised external and internal controls, respectively. External controls included healthy patients or patients who underwent non-diversion surgery; internal controls consisted of mucosa comparisons between diverted and proximal colons (singe time point) or longitudinal sampling of the colon in relation to faecal diversion or restoration, which provided temporal data. All studies, except Young et al. (2013), Baek et al. (2014), Beamish et al. (2017), Tominaga et al. (2021a, 2021b), Watanabe et al. (2021), sampled mucosal biopsies (among other methods) which are generally considered more representative of the mucosal microbiome. However, microbiome analysis methods differed with Beamish et al. (2017) and Baek et al. (2014) opting for PCR or culture-dependent methods instead of gene sequencing. It is also worth noting some studies such as Watanabe et al. ((2021) included other forms of dysbiosis measures such as histopathology and cytometry which provide information regarding intestinal health in addition to microbiome changes. Sample times varied significantly ranging from 1 to 40 months post-diversion. In summary, the studies included were generally small and heterogenous; therefore, a meta-analysis was not possible; we opted instead to perform a qualitative and narrative synthesis of the available data. Methods of DNA extraction, sequencing and analysis methods are summarised in Table 3.

**Table 2.** Characteristics of studies: Methods

References	Methods					
	Type of sample	Comparison/control	Sample time	Bowel prep	Analysis method
Beamish et al. (2017)	Mucosal Biopsy	Functional ileum biopsy compared against defunctioned ileum biopsy in same patient	During Ileostomy Reversal	None	16s rRNA qRT-PCR DGGE-PCR Histopathology
Tominaga et al. (2021a)	Mucosal Biopsy	Diverted colon biopsy in diversion group (before FMT) compared to faeces of healthy controls	6-40 months post-ileostomy	N/S	16S rRNA sequencing
Tominaga et al. (2021b)	Mucosal Biopsy	Diverted colon biopsy compared to proximal colon samples in same patient	1–40 months post-ileostomy	N/S	16S rRNA sequencing ELISA for SCFA and IgA
Baek et al. (2014)	Mucosal Brushings	Diverted colon brushings in diversion group compared to colon brushings in control group (surgical resection without diversion).	Pre-ileostomy reversal (2-3 months after primary surgery)	None	Plate Culture Targeted PCR of bacteria Histopathology
Young et al. (2013)	Mucosal Biopsy Mucosal Brushings Stool Aspirates	Biopsy, brush and aspirate samples in diversion group (for reversal) compared to healthy controls	2 weeks pre-reversal 2 weeks post-reversal 4 weeks post-reversal 8 weeks post-reversal	None	16s rRNA sequencing Culture and direct cell counts
Watanabe et al. (2021)	Mucosal Biopsy Mucosal Brushings	CD group: Biopsy and brushings in defunctioned ileum compared to functional ileum samples in same patient CRC group: Biopsy and brushings in defunctioned ileum compared to functional ileum samples in same patient	CD group: During temporary ileostomy and during closure (median time to closure: 21 months [IQR 3.8-43.5]) CRC group: During temporary ileostomy and during closure (median time to closure: 7 months [IQR 5.2-9])	N/S	qPCR and 16s rRNA IHC, TUNEL Assay & FC Histopathology

Table 2 summarising the methodology of included studies revealing significant heterogeneity between studies. Only relevant data pertaining to bowel diversion was extracted.

CD, Crohn’s disease; CRC, colorectal cancer; DC, diversion colitis; FC, flow cytometry; IHC, immunohistochemistry; N/S, not stated.

**Table 3.** Summary of methods used for specimen collection, DNA extraction, sequencing and analysis

References	DNA extraction	Sequencing	Analyses performed
Beamish et al. (2017)	**Mucosal Bacterial DNA Extraction** Total Genomic DNA extracted using QIAmp Cador pathogen minikit (Qiagen) **Luminal Bacterial DNA Extraction** Centrifuged in pathogen lysis tubes DNA Extraction with QIAmp UCP Pathogen minikit (Qiagen)	**Denaturation Gradient Gel Electrophoresis (DGGE) Profiling** Bacterial 16S V3 region **DGGE Band Excision & Sequencing** Sanger sequencing **16SrDNA qRT-PCR**	**Histological** Villous height measurement H&E sections scored for inflammation **Immunofluorescence PCNA Analysis** No. of PCNA positive intestinal epithelial cells (IEC) per crypt Percentage of proliferative to all nucleated cells **TUNEL Assay** No. of apoptotic cells per villous **Determination of Total Bacterial Load** Measured in qPCR comparing functional and defunctioned intestine
Tominaga et al. (2021a)	DNA extracted using a bead beating method and purified.	**PCR Amplification** Using TaKaRa Ex Taq Hot Start PCR mixture Bacterial 16S V3-4 regions **Illumina MiSeq sequencing**	**Endoscopic Evaluation and Symptoms** **Overall Diversity Analysis** Using QIIME software package to analyse alpha and beta diversity
Tominaga et al. (2021b)	DNA extracted using a bead beating method and purified.	**PCR Amplification** Using TaKaRa Ex Taq Hot Start PCR mixture Bacterial 16S V3-4 regions **Illumina MiSeq sequencing**	**Endoscopic Evaluation and Symptoms** **Overall Diversity Analysis** Using QIIME software package to analyse alpha and beta diversity **Intestinal SCFA and IgA**
Baek et al. (2014)	DNA extraction using DNA extraction kit (Intron Biotechnology)	**PCR Amplification on 11 bacterial genera** Using TaKaRa Ex Taq Hot Start PCR mixture	**Endoscopic Evaluation and Symptoms** **Overall Diversity Analysis** Bacterial species identified qualitatively
Young et al. (2013)	DNA extraction using bead beating method	**Amplification by PCR** Bacterial 16S V3-5 region Sequencing using Roche Titanium amplicon sequencing protocols	**Direct count of Microorganism density** **Screening for butyrate producing taxa**
Watanabe et al. (2021)	DNA extracted using a bead beating method and purified.	**Quantitative Real Time PCR** Using Thunderbird^®^ SYBR^®^ qPCR Mix (Toyobo Life Science) Bacterial 16S V1-2 region	**Immunohistochemistry and TUNEL Assay** Villous height measurement Crypt depth No. of goblet, Ki-67 and TUNEL cells No. of IFN-γ and IL-17 cells per high-power field **Isolation and flow cytometry analysis of Lamina Propria cells**

### Diversion and diversity

Five of the included 6 studies (excluding Baek et al., 2014) reported on diversity measures with results summarised in Table 4. Beamish et al. (2017) reported a reduction in total mucosal bacterial load (-62.4%) and DGGE band profiling (-5 bands) between diverted and proximal mucosa. Tominaga et al. (2021a) reported a decrease in alpha diversity (chao1 p<0.01, OTU p<0.01) and significant difference in beta diversity (Unifrac p<0.01) when comparing mucosal microbiome of DC patients to faeces of healthy controls. The mucosal–faecal comparison was not ideal; however, the authors presumably wanted to avoid subjecting healthy controls to unnecessary biopsies. Moreover, the findings of decreased alpha and beta diversity are consistent with other included studies such as Watanabe et al. (2021) which add validity. Interestingly, Tominaga et al. (2021b) who, like Beamish et al. (2017) compared microbiome compositions of the proximal and diverted colon reported no difference in alpha diversity (chao1 p=0.69, Shannon p=0.23) although the difference in beta diversity was significant (Unifrac p<0.05), signifying a difference in microbiome composition. It is worth noting however, that unlike Beamish et al. (2017) (n=34), this study was much smaller (n=8) and therefore may simply have been underpowered. Young et al. (2013) and Watanabe et al. (2021) were the only two studies utilising longitudinal sampling. The former found significantly decreased alpha diversity and reductions in viable cell counts in the diverted mucosa prior to ileostomy reversal compared to after. Surprisingly, the alpha diversity increased to the range of healthy control samples after 2 months post-reversal; however, the viable cell counts, though increased, remained lower than controls. Watanabe et al. (2021) similarly concluded that alpha diversity (chao1 p=0.001, Shannon p<0.001, OTU p=0.015) and mucosal bacterial load (p<0.01) were markedly reduced in the diverted colon compared to the proximal colon. Post-reversal analysis was not done with respect to the microbiome. In conclusion, there is limited but significant evidence that diversion and enteral nutrient deprivation reduces microbiome diversity, possibly predisposing patients to dysbiosis-related outcomes.

**Table 4.** Diversity changes following diversion

References	Alpha diversity	Beta diversity	Other diversity measures
Beamish et al. (2017)	N/S	N/S	Decreased Total mucosal bacterial load decreased 62.4% (p=0.0003) Mean DGGE profile reduction of ~5 bands (p< 0.04)
Tominaga et al. (2021a)	Decreased Chao1 (p<0.01) Observed OTU (p<0.01)	Decreased Weighted and unweighted Unifrac (p<0.01)	N/S
Tominaga et al. (2021b)	No difference Chao1 (p=0.69) Shannon Index (p=0.23)	Decreased Weighted and unweighted Unifrac (p<0.05)	N/S
Baek et al. (2014)	N/S	N/S	N/S
Young et al. (2013)	Pre-ileostomy reversal Decreased Shannon Index Post-ileostomy reversalIncreased Shannon Index	*N/S*	Pre-ileostomy reversal Decreased Reduction in direct and viable cell counts Post-ileostomy reversalIncreased Increase in direct and viable cell count (p=0.003)
Watanabe et al. (2021)	Pre-ileostomy reversal Decreased Chao1 (p=0.001) Shannon Index (p<0.001) Observed OTU (p=0.015) Post-ileostomy reversal N/S	N/S	Pre-ileostomy reversal Decreased Total mucosal bacterial load decreased (p<0.01) Post-ileostomy reversal N/S

Table 4 summarising microbiome diversity changes following diversion. Only significant increases or decreases reported. P values recorded together with diversity measure if available. Pre- and post-ileostomy reversal data reported where available.

DGGE, denaturing gradient gel electrophoresis; N/S, not stated; OTU, operational taxonomic unit.

### Phylum- and genus-specific changes

Five out of 6 studies (excluding Tominaga et al., 2021a) reported phylum- and genus-specific changes, which are summarised in Table 5. Increases or decreases were defined as at least one supporting study without any conflict.

**Table 5.** Effect of diversion on genus/phylum composition of the gut microbiome

Bacteria (genus/phylum)	Genus/phylum significance	Outcome	Supporting authors
Firmicutes	Unclear. Depends on species however increases in Firmicutes-Bacteroidetes ratios are generally indicative of dysbiosis (Lightner and Pemberton, 2017; Ferrie et al., 2020).	*Decreased*	Beamish et al. (2017); Young et al. (2013)
Bacteroidetes	Unclear. Depends on species however increases in Firmicutes-Bacteroidetes ratios are generally indicative of dysbiosis (Lightner and Pemberton, 2017; Ferrie et al., 2020).	*Unclear*	Young et al. (2013);Beamish et al. (2017)
Proteobacteria	Generally harmful. Contains many pathogenic genera and increase is a signature of dysbiosis (Štšepetova et al., 2022).	*Increased*	Beamish et al. (2017)
*Lactobacillus* (Firmicutes)	Beneficial. SCFA producing and have a role in intestinal function and immunity, as well as epithelial integrity maintenance (Azad et al. 2018).	*Decreased*	Tominaga et al. (2021b); Baek et al. (2014)
*Bifidobacterium* (Actinomycetes)	Beneficial. SCFA producing and have a role in intestinal function and immunity, as well as epithelial integrity maintenance (Azad et al. 2018).	*Decreased*	Baek et al. (2014)
*Granulicatella* (Firmicutes)	Commensal. Part of normal flora but translocation is linked to serious infections (Sędzikowska and Szablewski, 2021).	*Decreased*	Tominaga et al. (2021b); Watanabe et al. (2021)
*Clostridium* (Firmicutes)	Unclear. Depends on species. Predominant gut commensal. Some are SCFA producing while others are pathogenic (Ferrie et al., 2020).	*Decreased*	Beamish et al. (2017)
*Escherichia* (Proteobacteria)	Commensal. Part of normal flora but includes several pathogenic species. Increase is generally harmful (Brown et al., 2014; Ferrie et al., 2020).	*Decreased*	Beamish et al. (2017)
*Streptococcus* (Firmicutes)	Unclear. Depends on species. Some are SCFA producing while others are pathogenic (Ferrie et al., 2020; Martinson and Walk, 2020).	*Decreased*	Beamish et al. (2017)
*Spirosoma* (Bacteroidetes)	Unclear. Limited data, however, some animal studies note increase in diarrheal states (Roux et al., 2022).	*Increased*	Beamish et al. (2017)
*Klebsiella* (Proteobacteria)	Generally harmful. Increase is linked to infection and disease (Xi et al., 2021).	*Decreased*	Baek et al. (2014)
*Pseudomonas* (Proteobacteria)	Generally harmful. Not considered commensal. Increase is linked to dysbiosis (Wu et al., 2020).	*Decreased*	Baek et al. (2014)
*Enterococcus* (Firmicutes)	Generally harmful. Increase is linked to infection and disease (Pettigrew et al., 2018; Ferrie et al., 2020).	*Decreased*	Baek et al. (2014)
*Staphylococcus* (Firmicutes)	Generally harmful. Colonisation and overgrowth are linked to infection and disease (Zhang et al., 2015).	*Decreased*	Baek et al. (2014)
*Anaerococcus* (Firmicutes)	Potentially harmful. Limited data but increases associated with malnutrition and coeliac’s (Raineri et al., 2021; Rodríguez-Padilla et al., 2021).	*Increased*	Tominaga et al. (2021b)
*Corynebacterium* (Actinomycetes)	Potentially harmful. Some species associated with infection such as diphtheria (Lopetuso et al., 2023).	*Increased*	Tominaga et al. (2021b)
*Peptoniphilus* (Firmicutes)	Commensal. However, translocation and overgrowth linked to blood and tissue infections (Gowen et al., 2023).	*Increased*	Tominaga et al. (2021b)
*Porphyromonas* (Bacteroidetes)	Potentially harmful. Increase has been linked to pro-inflammatory chronic states (Lopetuso et al., 2023).	*Increased*	Tominaga et al. (2021b)
*Bacteroides* (Bacteroidetes)	Generally Beneficial. SCFA producing with key homeostasis and functional roles in intestine (Ralls et al., 2013).	*Unclear*	Beamish et al. (2017)
Actinomyces (Actinomycetes)	Potentially harmful. Several species associated with actinomycosis infections (Jandhyala, 2015).	*Unclear*	Watanabe et al. (2021)

Table 5 highlighting genus/phyla specific changes associated with diversion together with their potential significance. *Increases/Decreases* were defined as at least 1 supporting study among the included studies without any conflict with the others. *Unclear* was defined as conflicting data within a single study or between studies. Only significant results reported.

Table format and analysis adapted from Ferrie et al. (2020).

At a phylum level, both Beamish et al. (2017) and Young et al. (2013) reported significantly decreased (21% reduction, p= 0.02) Bacillota compositions in the diverted colon. The former also reported an increase in Pseudomonadota, (6.9%, p=0.05) but found significant variation in Bacteroidia composition, as opposed to Young et al. (2013) who described a decrease in Bacteroidia. While the significance of Bacteroidia and Bacillota changes are unclear without species-specific information, Pseudomonadota increases are strongly indicative of a microbial “dysbiosis signature” due to its abundance of pathogenic genera (Shin et al., 2015). Thus, Bacillota reduction with concomitant increases in Pseudomonadota suggests dysbiosis in the diverted colon (Shin et al., 2015).

At a genus level, Baek et al. (2014) found diversion correlated with decreases in *Lactobacillus* (p=0.038) and *Bifidobacterium* (p<0.001), with Tominaga et al. (2021b) also finding a *Lactobacillus* decline (p<0.05). Both bacteria are regarded as beneficial due to their roles in metabolism, intestinal immunity and epithelial maintenance, with decreases associated with dysbiosis. Könönen and Wade (2015) Interestingly, Baek et al. (2014) additionally found *Bifidobacterium* as the only genus significantly and inversely correlated with the severity of diversion colitis in patients, highlighting its potential clinical significance. Beamish et al. (2017) also reported a 36.3% decrease in *Clostridium* abundance in the diverted colon. While the significance of this is debatable without species-level information (as some can be pathogenic), *Clostridia* are nevertheless a predominant cluster of gut commensals and are generally SCFA-producing (Guo et al., 2020). Such a large decrease inevitably affects microbiome homeostasis, potentially leading to dysbiosis in the diverted colon.

Other less-specific changes in the diverted colon were also recorded. Tominaga et al. (2021b) found increased levels of *Corynebacterium* (p<0.01), *Peptoniphilus* (p<0.05), *Anaerococcus* (p<0.05) and *Porphyromonas* (p<0.01). These genera have been associated with haematogenous or tissue infections when translocated or overgrown (Brown et al., 2014; Tidjani Alou et al., 2016; Sędzikowska and Szablewski, 2021; Štšepetova et al., 2022). However, their role in intestinal inflammation is less clear at this stage. *Porphyromonas* for example has been linked to oral infections which does not necessarily translate to intestinal inflammation (Sędzikowska and Szablewski, 2021). Other non-specific changes reported in the diverted colon include decreases in *Escherichia (-9%)*, *Streptococci (-36.3%)* and increases in *Spirosoma* (+27%) as reported by Beamish et al. (2017); as well as decreases in *Klebsiella (p<0.001)*, *Pseudomonas (p<0.015)*, *Enterococci (p<0.001)* and *Staphylococci* (p<0.038) by Baek et al. (2014) Most of these bacteria are potential pathobionts and these changes when viewed simplistically, could be seen as a positive outcome from diversion (Zhang et al., 2015; Pettigrew et al., 2018; Martinson and Walk, 2020; Wu et al., 2020; Raineri et al., 2021; Xi et al., 2021; Roux et al., 2022). However, stability and balance of the microbiome appear to be more important determinants of gut health compared to the absence of specific pathogenic genera.

Following ileostomy reversal, Young et al. (2013) reported increases in butyrate metabolism and potentially beneficial SCFA-producing microorganisms such as *Acidaminococcus* and *Coprococcus* up to 60 days post-operatively, indicating some level of reversibility; however, the overall microbiome profiles remained different, and its significance was not explored further in these studies.

### Other dysbiosis changes

Diversion and microbiome changes were also associated with immunological and functional dysregulation. Both Beamish et al. (2017) (p=0.0004, p=0.01) and Watanabe et al. (2021) (p<0.01, p<0.01) found villous atrophy and reduced crypt cell proliferation in the diverted colon. In addition, Watanabe et al. (2021) also found decreased CD3+ (p=0.037), IL17+ (p=0.002) and IFN-G+ (p=0.013) T-cells signifying immune dysregulation. More importantly, Tominaga et al. (2021b) found decreased SCFA levels (p<0.05) in the diverted colon which adds further weight that the intestinal environment is lacking SCFA-producing bacteria. Interestingly, following ileostomy reversal, Watanabe et al. (2021) reported restoration in villous height, goblet cells and immune cells back to functional ileum levels indicating reversibility of these changes post-ileostomy reversal.

### Quality assessment

The quality of papers ranged from scores of 6.5/10 to 9.5/10, and limitations were generally due to sampling methodology or suboptimal controls and outcome measurement. Importantly, different studies also used different methods to characterise the gut microbiota. Baek et al. used quantitative PCR and Beamish used DGGE, while Young, Watanabe and Tominaga (a and b) used 16S rRNA sequencing (Young et al., 2013; Baek et al., 2014; Beamish et al., 2017; Watanabe et al., 2021; Tominaga et al., 2021a, 2021b). Additionally, within the 16S rRNA-based studies, different regions were sequenced (Young (V3-V5), Watanabe (V1-V2), Tominaga (a and b) V3-V4), which can lead to technical compositional biases between studies.

A further limitation of these studies was the lack of longitudinal assessment, which could mean that the influence of underlying disease (i.e. malignancy or IBD) causing dysbiosis may not be fully characterised.

### Discussion

This systematic review included 6 studies that examined the impacts of faecal diversion on the diverted gut microbiome.

Our review suggests that faecal diversion is associated with a decrease in microbiome diversity, as well as microbiome and intestinal changes suggesting dysbiosis and dysfunction. The loss of diversity together with SCFA-producing bacteria is consistent with inflammatory states such as in IBD as confirmed by other studies (Ferrie et al., 2020). While the direction of cause and effect is not immediately clear, a recent RCT found that probiotic stimulation of the diverted bowel loops significantly improved clinical and histological signs of severe and moderate DC in 100% and 88% of patients, respectively (Rodríguez-Padilla et al., 2021). This points towards an active role of the microbiome in modulating immunity and function rather than simply being a biomarker of dysbiosis. More importantly, this study successfully utilised *Lactobacillus* and *Bifidobacterium* in its probiotic therapy, which is again consistent with our findings of decreased *Lactobacillus* and *Bifidobacterium* in DC patients. Similarly, Tominaga et al. (2021a) performed autologous FMT on 5 patients with severe DC, achieving 100% subsequent remission. Both study authors conclude that the use of probiotics and FMT, respectively, are effective and safe treatments for DC indicating the importance of the microbiome in the development of future treatments. While our study reaffirmed the partial reversibility of microbiome and intestinal changes following the reversal of faecal diversion, thus supporting it as a treatment for DC; these experimental treatments open future possibilities for treatment options of persistent DC or where reversal is contraindicated.

While the treatment of DC with microbiome modulation has not yet been truly investigated, recent studies show promising results when dealing with inflammatory bowel disease (IBD). Recently, an international Rome consensus was published, acknowledging the role of the gut microbiome in the development of IBD and the utility of faecal microbiota transplant (FMT) as a viable treatment option for mild-to-moderate ulcerative colitis (UC) on a case-by-case basis, although this has not been proven in Crohn’s disease (CD) (Lopetuso et al., 2023). Instead, assessments of the microbiome could be used to monitor disease activity.

Use of probiotics and prebiotics has also been mooted as a potential longer-term mechanism of modulating the microbiome. The theory of replenishing organisms which are lacking in a specific disease has shown promise in the treatment of IBD (Gowen et al., 2023). However, the specific dose and species of probiotic bacterium used to treat a specific condition have yet to be established and remain an area of ongoing research (Lopetuso et al., 2023).

Our study results are also relevant in the context of prolonged enteral starvation and loop stoma reversal. Ralls et al. found, for example, that total parental nutrition (TPN) use was associated with decreased microbial diversity in humans, as well as a decrease and increase in Bacillota and Pseudomonadota, respectively, similar to the findings by Ralls et al. (2013), Beamish et al. (2017). These changes were exaggerated with prolonged TPN, and associated with increased anastomotic post-operative leakage and infection (Ralls et al., 2013). The potential link between anastomotic leakage and microbiome dysbiosis is important as it raises the question of whether enteral supplementation of the affected colon (probiotic, SCFA, faecal), especially in the cases of prolonged diversion or TPN, prior to reversal would reduce risk of anastomotic leakage as proposed by Beamish et al. (2017). The results of our study indeed point towards dysbiosis, dysfunction and inflammation that predisposes leakage, thus supporting this recommendation for future trials.

Limitations of this review include the small number of relevant studies and the heterogeneity in methodology between them. Even though we attempted to control for the quality of included studies, the heterogeneity in comparators, sampling time and analysis techniques limits comparison between studies. Previous reviews have shown, for example, that faecal samples can significantly differ from mucosal samples (Jandhyala, 2015). Moreover, the sample timings also differed significantly between studies, raising the possibility that some patients may not have been given enough time for the diverted microbiome communities to stabilise before sampling. Bowel preparation use was also unclear in 3 of the 6 studies.

Furthermore, a recent systematic review concluded that even sequencing kits and sample storage conditions may affect microbiome composition (Ferrie et al., 2020). Storage temperature, for example, may alter the sequenced Bacteroidia: Bacillota ratio (Ferrie et al., 2020). Given the heterogeneity of the included studies and the difficulty of pooled analysis, a standardised protocol for sampling methods, sites and timing, as well as analysis methods in the context of future, adequately powered observational studies or trials are required as recommended by Ferrie et al. (2020).

It is important to note that other components of the gastrointestinal tract such as the virome and mycobiome, which are outside the scope of this review, also contribute to the diversity, health and function of the colon.

## Conclusion

This systematic review identified 6 relevant papers examining the impacts of faecal diversion on the diverted gut microbiome. Five of these papers reported significant decreases in microbial diversity after diversion, in addition to phyla and genus-specific changes such as a loss of SCFA-producing genera that support a dysbiosis profile. Moreover, additional immunological and histological evidence support a dysregulated and dysfunctional intestinal environment associated with the microbiome changes.

Restoration of the faecal stream was then associated with improvements in the dysbiosis profile and intestinal function. Novel techniques such as probiotic stimulation and FMT in the efferent limb have shown promise in the treatment of dysbiosis outcomes such as DC. Furthermore, in the context of prolonged starvation or deprivation as is the case for diversion, supplementation of the affected colon may, in theory, reduce anastomotic leakage prior to loop stoma reversal or other forms of reconstructive bowel surgery. These techniques may even have a role to play in improving functional outcomes, but require further investigation to determine their roles and clinical applicability.

The greatest limitations of these studies appear to be scale and power, as the processes involved in sample collection and analysis can be complex and require specific technical ability, due to the high level of interindividual variability and diversity within the colonic microbiome. This therefore highlights the need for further standardised collaborative studies, and the value of systematic reviews to provide context for further advancements into a field becoming increasingly relevant to modern day clinical practice.

## Data Availability

Data from this review can be made available upon reasonable request from the corresponding author.

## List of abbreviations

CA: Cancer
CD: Crohn’s Disease
CENTRAL: Cochrane Central Register of Controlled Trials
CRC: Colorectal Cancer
DC: Diversion Colitis
DGGE: Denaturing Gradient Gel Electrophoresis
FMT: Faecal Microbial Transplant
FC: Flow Cytometry
GI/GIT: Gastrointestinal/Gastrointestinal Tract
IBD: Inflammatory Bowel Disease
IHC: Immunohistochemistry
IPAA: Ileal Pouch Anal Anastomosis
LARS: Lower Anterior Resection Syndrome
NRS: Non-Randomised Controlled Study
OTU: Operational Taxonomic Unit
PCR/qPCR: Polymerase Chain Reaction/quantitative Polymerase Chain Reaction
PRISMA: Preferred Reporting Items for Systematic Reviews and Meta-analyses
RCT: Randomised Controlled Trial
SCFA (s): Short-chain Fatty Acid (s)
TPN: Total Parenteral Nutrition
UC: Ulcerative Colitis

## References

[r47] Azad M, Sarker M, Li T and Yin J (2018) Probiotic Species in the Modulation of Gut Microbiota: An Overview. BioMed Research International 1–8. 10.1155/2018/9478630 PMC5964481 29854813

[r1] Baek S-J, Kim S-H, Lee C-K, Roh K-H, Keum B, Kim C-H and Kim J (2014) Relationship between the severity of diversion colitis and the composition of colonic bacteria: A prospective study. Gut and Liver 8(2), 170–176. 24672659 10.5009/gnl.2014.8.2.170 PMC3964268

[r2] Beamish E, Johnson J, Shaw E, Scott N, Bhowmick A and Rigby R (2017) Loop ileostomy-mediated fecal stream diversion is associated with microbial dysbiosis. Gut Microbes 8(5), 467–478. 28622070 10.1080/19490976.2017.1339003 PMC5628638

[r3] Berg G, Rybakova D, Fischer D, Cernava T, Vergès M, Charles T, et al. (2020) Microbiome definition re-visited: Old concepts and new challenges. Microbiome 8(1), 103. 10.1186/s40168-020-00875-0. Erratum in: Microbiome. 2020 8(1), 119. PMID: 32605663. PMCID: PMC7329523. 32605663 PMC7329523

[r4] Brown K, Church D, Lynch T and Gregson D (2014) Bloodstream infections due to Peptoniphilus spp.: Report of 15 cases. Clinical Microbiology and Infection 20(11), O857–O860. 24773457 10.1111/1469-0691.12657 PMC4304329

[r5] Deschasaux M, Bouter K, Prodan A, Levin E, Groen A, Herrema H, et al. (2018) Depicting the composition of gut microbiota in a population with varied ethnic origins but shared geography. Nature Medicine 24(10), 1526–1531. 10.1038/s41591-018-0160-1 30150717

[r6] Doré J, Simrén M, Buttle L and Guarner F (2013) Hot topics in gut microbiota. United European Gastroenterology Journal 1(5), 311–318. 24917977 10.1177/2050640613502477 PMC4040776

[r7] Dou X, Gao N, Yan D and Shan A (2020) Sodium butyrate alleviates mouse colitis by regulating gut microbiota Dysbiosis. Animals 10(7), 1154. 32645998 10.3390/ani10071154 PMC7401615

[r8] Ferrie S, Webster A, Wu B, Tan C and Carey S (2020) Gastrointestinal surgery and the gut microbiome: A systematic literature review. European Journal of Clinical Nutrition 75(1), 12–25. 32661352 10.1038/s41430-020-0681-9

[r9] Gacesa R, Kurilshikov A, Vich Vila A, Sinha T, Klaassen MAY, Bolte LA, Andreu-Sánchez S, Chen L, Collij V, Hu S, Dekens JAM, Lenters VC, Björd JR, Swarte JC, Swertz MA, Jansen BH, Gelderloos-Arends J, Jankipersadsing S, Hofker M, … Weersma RK (2022) Environmental factors shaping the gut microbiome in a Dutch population. Nature 7907, 732–739. 10.1038/s41586-022-04567-7 35418674

[r10] Gowen R, Gamal A, Di Martino L, McCormick TS and Ghannoum MA (2023) Modulating the microbiome for Crohn’s disease treatment. Gastroenterology 164(5), 828–840. 10.1053/j.gastro.2023.01.017. 36702360 PMC10152883

[r11] Guo P, Zhang K, Ma X and He P (2020) Clostridium species as probiotics: Potentials and challenges. Journal of Animal Science and Biotechnology 11(1), 24. 10.1186/s40104-019-0402-1. PMID: 32099648; PMCID: PMC7031906. 32099648 PMC7031906

[r12] Harig J, Soergel K, Komorowski R and Wood C (1989) Treatment of diversion colitis with short-chain-fatty acid irrigation. New England Journal of Medicine 320(1), 23–28. 2909876 10.1056/NEJM198901053200105

[r13] Harries R, Ansell J, Codd R and Williams G (2017) A systematic review of Clostridium difficile infection following reversal of ileostomy. Colorectal Disease 19(10), 881–887. 28872758 10.1111/codi.13873

[r14] Jandhyala S (2015) Role of the normal gut microbiota. World Journal of Gastroenterology 21(29), 8787. 26269668 10.3748/wjg.v21.i29.8787 PMC4528021

[r15] Joanna Briggs Institute (2022) Critical appraisal tools. Available at https://jbi.global/critical-appraisal-tools (accessed 15 August 2022).

[r16] Könönen E and Wade W (2015) Actinomyces and related organisms in human infections. Clinical Microbiology Reviews 28(2), 419–442. 25788515 10.1128/CMR.00100-14 PMC4402957

[r17] Lightner A and Pemberton J (2017) The role of temporary fecal diversion. Clinics in Colon and Rectal Surgery 30(3), 178–183. 28684935 10.1055/s-0037-1598158 PMC5498160

[r18] Lopetuso LR, Deleu S, Godny L, Petito V, Puca P, Facciotti F, Sokol H, Ianiro G, Masucci L, Abreu M, Dotan I, Costello SP, Hart A, Iqbal TH, Paramsothy S, Sanguinetti M, Danese S, Tilg H, Cominelli F, Pizarro TT, Armuzzi A, Cammarota G, Gasbarrini A, Vermeire S and Scaldaferri F (2023) The first international Rome consensus conference on gut microbiota and faecal microbiota transplantation in inflammatory bowel disease. Gut 72(9), 1642–1650. 10.1136/gutjnl-2023-329948 37339849 PMC10423477

[r19] Martinson J and Walk S (2020) Escherichia coli residency in the gut of healthy human adults. EcoSal Plus 9(1), 10.1128/ecosalplus.esp-0003-2020. PMID: 32978935; PMCID: PMC7523338. PMC7523338 32978935

[r20] Pettigrew M, Gent J, Kong Y, Halpin A, Pineles L, Harris A, et al. (2018) Gastrointestinal microbiota disruption and risk of colonization with Carbapenem-resistant Pseudomonas aeruginosa in intensive care unit patients. Clinical Infectious Diseases 69(4), 604–613. 10.1093/cid/ciy936 PMC6669284 30383203

[r21] Pieniowski E, Nordenvall C, Palmer G, Johar A, Tumlin Ekelund S, Lagergren P, et al. (2020) Prevalence of low anterior resection syndrome and impact on quality of life after rectal cancer surgery: Population-based study. BJS Open 4(5), 935–942. 32530135 10.1002/bjs5.50312 PMC7528525

[r22] Radjabzadeh D, Boer C, Beth S, van der Wal P, Kiefte-De Jong J, Jansen M, et al. (2020) Diversity, compositional and functional differences between gut microbiota of children and adults. Scientific Reports 10(1), 1040. 10.1038/s41598-020-57734-z. 31974429 PMC6978381

[r23] Raineri E, Altulea D and van Dijl J (2021) Staphylococcal trafficking and infection—From ‘nose to gut’ and back. FEMS Microbiology Reviews 46(1):fuab041. 10.1093/femsre/fuab041. PMCID: PMC8767451. PMC8767451 34259843

[r24] Ralls M, Miyasaka E and Teitelbaum D (2013) Intestinal microbial diversity and perioperative complications. Journal of Parenteral and Enteral Nutrition 38(3), 392–399. 23636012 10.1177/0148607113486482 PMC4183124

[r25] Rao K and Safdar N (2015) Fecal microbiota transplantation for the treatment of Clostridium difficile infection. Journal of Hospital Medicine 11(1), 56–61. 26344412 10.1002/jhm.2449 PMC4908581

[r26] Remzi F (2017) Fecal diversion in patients with Crohn’s disease. Gastroenterology & Hepatology 15(8), 431–433. PMC6771030 31592243

[r27] Rodríguez-Padilla Á, Morales-Martín G, Pérez-Quintero R, Rada-Morgades R, Gómez-Salgado J and Ruiz-Frutos C (2021) Diversion colitis and probiotic stimulation: Effects of bowel stimulation prior to ileostomy closure. Frontiers in Medicine 8, 654573. 10.3389/fmed.2021.654573. PMID: 34249962; PMCID: PMC8267790 34249962 PMC8267790

[r28] Rolhion N and Chassaing B (2016) When pathogenic bacteria meet the intestinal microbiota. Philosophical Transactions of the Royal Society B: Biological Sciences 371(1707), 20150504. 10.1098/rstb.2015.0504 PMC5052746 27672153

[r29] Roux E, Nicolas A, Valence F, Siekaniec G, Chuat V, Nicolas J, et al. (2022) The genomic basis of the Streptococcus thermophilus health-promoting properties. BMC Genomics 23(1), 210. 10.1186/s12864-022-08459-y. PMID: 35291951; PMCID: PMC8925076. 35291951 PMC8925076

[r30] Sędzikowska A and Szablewski L (2021) Human gut microbiota in health and selected cancers. International Journal of Molecular Sciences 22(24), 13440. 34948234 10.3390/ijms222413440 PMC8708499

[r31] Sender R, Fuchs S and Milo R (2016) Revised estimates for the number of human and bacteria cells in the body. PLOS Biology 14(8), e1002533. 10.1371/journal.pbio.1002533. PMID: 27541692; PMCID: PMC4991899. 27541692 PMC4991899

[r32] Shin N, Whon T and Bae J (2015) Pseudomonadota: Microbial signature of dysbiosis in gut microbiota. Trends in Biotechnology 33(9), 496–503. 26210164 10.1016/j.tibtech.2015.06.011

[r33] Shreiner A, Kao J and Young V (2015) The gut microbiome in health and in disease. Current Opinion in Gastroenterology 31(1), 69–75. 25394236 10.1097/MOG.0000000000000139 PMC4290017

[r34] Štšepetova J, Simre K, Tagoma A, Uibo O, Peet A, Siljander H, et al. (2022) Maternal breast milk microbiota and immune markers in relation to subsequent development of celiac disease in offspring. Scientific Reports 12(1), 6607. 10.1038/s41598-022-10679-x. Erratum in: *Sci Rep.* 2022;12(1), 7875. PMID: 35459889; PMCID: PMC9033794. 35459889 PMC9033794

[r35] Ten Hove J, Bogaerts J, Bak M, Laclé M, Meij V, Derikx L, et al. (2018) Malignant and nonmalignant complications of the rectal stump in patients with inflammatory bowel disease. Inflammatory Bowel Diseases 25(2), 377–384. 10.1093/ibd/izy253 30085111

[r36] Tidjani Alou M, Khelaifia S, Michelle C, Andrieu C, Armstrong N, Bittar F, et al. (2016) Anaerococcus rubiinfantis sp. nov., isolated from the gut microbiota of a Senegalese infant with severe acute malnutrition. Anaerobe 40, 85–94. 27328611 10.1016/j.anaerobe.2016.06.007

[r37] Tominaga K, Tsuchiya A, Mizusawa T, Matsumoto A, Minemura A, Oka K, Takahashi M, Yoshida T, Kojima Y, Ogawa K, Kawata Y, Nakajima N, Kimura N, Abe H, Setsu T, Takahashi K, Sato H, Ikarashi S, Hayashi K, Mizuno KI, Yokoyama J, Tajima Y, Nakano M, Shimada Y, Kameyama H, Wakai T and Terai S (2021a) Utility of autologous fecal microbiota transplantation and elucidation of microbiota in diversion colitis. DEN Open 2(1), e63. 10.1002/deo2.63. PMID: 35310733; PMCID: PMC8828251. 35310733 PMC8828251

[r38] Tominaga K, Tsuchiya A, Mizusawa T, Matsumoto A, Minemura A, Oka K, et al. (2021b) Evaluation of intestinal microbiota, short-chain fatty acids, and immunoglobulin a in diversion colitis. Biochemistry and Biophysics Reports 25, 100892. 33458259 10.1016/j.bbrep.2020.100892 PMC7797511

[r39] Valdes A, Walter J, Segal E and Spector T (2018) Role of the gut microbiota in nutrition and health. BMJ 361, k2179. 29899036 10.1136/bmj.k2179 PMC6000740

[r40] Watanabe Y, Mizushima T, Okumura R, Fujino S, Ogino T, Miyoshi N, et al. (2021) Fecal stream diversion changes intestinal environment, modulates mucosal barrier, and attenuates inflammatory cells in Crohn’s disease. Digestive Diseases and Sciences 67(6), 2143–2157. 34041649 10.1007/s10620-021-07060-9

[r41] Whelan R, Abramson D, Kim D and Hashmi H (1994) Diversion colitis. Surgical Endoscopy 8(1), 19–24. 8153859 10.1007/BF02909487

[r42] Wu T, Xu F, Su C, Li H, Lv N, Liu Y, et al. (2020) Alterations in the gut microbiome and cecal metabolome during Klebsiella pneumoniae-induced Pneumosepsis. Frontiers in Immunology 11, 1331. 10.3389/fimmu.2020.01331. PMID: 32849494; PMCID: PMC7411141. 32849494 PMC7411141

[r43] Xi L, Song Y, Han J and Qin X (2021) Microbiome analysis reveals the significant changes in gut microbiota of diarrheic Baer’s Pochards (Aythya baeri). Microbial Pathogenesis 157, 105015. 34062226 10.1016/j.micpath.2021.105015

[r44] Young VB, Raffals LH, Huse SM, Vital M, Dai D, Schloss PD, Brulc JM, Antonopoulos DA, Arrieta RL, Kwon JH, Reddy KG, Hubert NA, Grim SL, Vineis JH, Dalal S, Morrison HG, Eren AM, Meyer F, Schmidt TM, Tiedje JM, Chang EB and Sogin ML (2013) Multiphasic analysis of the temporal development of the distal gut microbiota in patients following ileal pouch anal anastomosis. Microbiome 1(1), 9. 10.1186/2049-2618-1-9. PMID: 24451366; PMCID: PMC3971607. 24451366 PMC3971607

[r45] Zhang Y, Li S, Gan R, Zhou T, Xu D and Li H (2015) Impacts of gut bacteria on human health and diseases. International Journal of Molecular Sciences 16(12), 7493–7519. 25849657 10.3390/ijms16047493 PMC4425030

[r46] Zhernakova A, Kurilshikov A, Bonder M, Tigchelaar E, Schirmer M, Vatanen T, et al. (2016) Population-based metagenomics analysis reveals markers for gut microbiome composition and diversity. Science 352(6285), 565–569. 27126040 10.1126/science.aad3369 PMC5240844

